# Psoas Abscess in Women1Read at the thirty-first annual meeting of the Medical Association of Central New York, at Auburn, October 18, 1898.

**Published:** 1898-12

**Authors:** Ely Van De Warker

**Affiliations:** Syracuse, N. Y.


### PSOAS ABSCESS IN WOMEN.[1]

By ELY VAN DE WARKER, M. D., Syracuse, N. Y.

THE classical idea of the psoas abscess is a pus accumulation, usually of spinal origin and confined to the sheath of the muscle and external to the peritoneum. The psoas abscess of women is almost without exception of pelvic origin and invades the aponeurosis of the muscle by extension in the direction of the least resistance, and like the classical form is always external to the peritoneum. It is a product of cellulitis and when it attains the level of the psoas is almost without exception a pus accumulation of great extent and serious import. In spite of the intolerance of the so-called pelvic surgeon of the possibility of pelvic cellulitis, the observing, scientific gynecologist is not only prepared to recognise its existence, but also to realise its serious character. There may be several points of origin within the pelvis. It may begin as a phlegmon of the broad ligament with extension to the iliac fossa, or as an extension from the fossa *de novo*, or from a recto-ischiatic abscess.

As a useful demonstration of the long duration of the cases and the enormous accumulation of pus that may result, as well as the difficulty of the diagnosis, I have one case that I would like to present in detail:

I was called by Dr. Dewitt, of Oswego, to see Mrs. B., married, aged 47 years, and never pregnant. The early history is unknown so far as explaining the condition as observed by me. Dr. Dewitt had charge of the case for two months, during which the conditions were unchanged. The examination showed a tumor filling the left iliac fossa, extending up to the ribs and reaching half way to the umbilicus toward the right. Palpation elicited an obscure fluctuation, equally diffused over the mass. Vaginal examination demonstrated that the mass extended downward into the pelvis on the left side, reaching to within half an inch of

1. Read at the thirty-first annual meeting of the Medical Association of Central New York, at Auburn, October 18, 1898.

the middle line in the posterior cul-de-sac. It was prolonged downward into the lateral vaginal wall, which was fixed to the bony wall of the pelvis. On bimanual palpation a distinct impact could be imparted to the vaginal tumor. Inspection of the vagina showed the rugæ obliterated with the surface smooth and shining. The left leg from the body to the foot was greatly swollen, measuring more than twice the circumference of the right leg. The thigh was half flexed upon the body and the leg upon the thigh. Any attempt to straighten the limb caused extreme pain as well as any effort to move in the bed. The patient made no complaint of abdominal pain or tenderness, all the pain being referred to the leg The general appearance of the patient was haggard and emaciated, appetite poor and sleep impossible except under opiates. The temperature, since coming under Dr. Dewitt's care, was rarely elevated above 100° and occasionally sub-normal in the morning. Urine gave a small amount of albumin and a few granular casts. Menstruation had ceased at the beginning of her illness, at the time of the examination, about eighteen months. The history, as given by the patient, threw but little light upon the case. She had had for several months, in the early part of the case, severe pain upon the left side of the pelvis, but with no enlargement that she was aware of or to which her attention was called by her physician. About a year previous to my visit the leg began to swell and became very tender and painful. She was treated then for inflammatory rheumatism. So far as her medical attendant knew, no diagnosis of pelvic disease had ever been made by the several physicians who had been in attendance. I stated in my opinion that the abdominal mass was of pelvic origin, that the swelling of the leg was due to obstruction of the venous circulation in the left pelvic hemisphere. As to the character of the contents I was unable to state. The history, so far as the absence of temperature and the prolonged history, appeared contrary to the theory of a pus content; with that explanation of her condition I feared malignancy. To clear up the obscure points I advised an exploratory incision. On this Dr. Dewitt concurred and in a few days I received notice to go to Oswego and operate. On September 12, 1895, I did the operation, assisted by Drs. Dewitt and Dowde.

The mass was explored through a short incision in the middle line. The mass was confined by a thin membrane to the left iliac fossa and the left abdominal cavity. The thin membrane was the peritoneum, which was reflected centrally half way to the umbilicus, carrying the colon and its mass to the right to an equal extent. It was evident that the accumulation was extraperitoneal and that no part of the peritoneal cavity could be brought to the middle line for a safe opening. An incision was made on a line perpendicular to the left anterior superior spine of the ileum. A large amount of pus, yellow and odorless, escaped and a drainage tube inserted. The escape of pus was enormous for many days. The patient gradually sank from exhaustion and died in about two weeks.

Upon this case I would like the privilege of making a few comments concerning the nature of these cases. They define a distinct group, and one peculiar to women. The psoas abscess of the general surgeon originates from a psoitis, or by implication from the vertebra, while in women we may say that, excluding caries of the vertebra, it never originates within the sheath of the muscle, but by extension from the intrapelvic cellular spaces. It is the product of pelvic cellulitis, pure and simple.

It is interesting to inquire why the psoas form of abscess may result, not from an extension of the cellulitis, but from the extension of a pelvic pus accumulation along lines of the least resistance. How this may occur is well shown in the experiments of König. He made injections beneath the peritoneum near the ovary. The fluid made its way along the psoas and iliacus muscles and diffused itself into that side of the pelvis; injected into the lateral (broad) ligament, close to the upper and anterior part of the cervix, it traversed the side of the pelvis, passed along the round ligament toward Poupart's ligament and thence into the iliac fossa; injected into the broad ligament, near the upper part of the cervix, but posterior to it, it filled the cellular spaces of the posterior and lateral part of the pelvis and thence passed along the psoas and iliac muscles. This demonstrates the continuity of the various areas of subperitoneal cellular tissue and also the manner in which a pelvic abscess becomes a psoas collection of pus.

The diagnosis of the condition is difficult. The collection is so deeply seated that fluctuation is difficult to detect. Temperature as an indication of pus may fail to be present. There are three conditions which, when present, clearly point out the fact that psoas and iliac regions are affected. These are edema of the leg, retraction of the thigh of the affected side, and extension downward of the cellular infiltration into the vaginal wall. Edema of the leg is an anatomical symptom of value. It has never, within my observation, been a phlebitis, but is due to pressure upon the venous system of the pelvis, which may be associated with a pus collection in the iliac fossa, but usually implies an implication of the iliac and psoas muscles.

Another condition very systematic is retraction of the thigh of the side involved. There are very evident anatomical reasons why retraction of the thigh is such an unmixed symptom of the pelvic cellulitis, with or without concurrent inflammation of the pelvic peritoneum. I have already referred to the direct route and interdependence between all the extensive spaces of the subperitoneal cellular tissue. Passing through these areas are the two important members of the machinery of locomotion. The psoas and iliacus muscles with their widely radiated origin are intimately connected with this great expansion of subperitoneal cellular tissue. The peritoneum over the anterior surface of the iliacus is practically the covering of the cellular phlegmon of the iliac fossa. The upper reaches of the psoas are not so directly exposed to the invasion of the cellular inflammation, but in the horizontal position, in which most of these cases are, pus may escape upward in the line of the least resistance. This is the mechanism of retraction of the leg, a symptom I have never seen attending pelvic peritonitis, unless associated with intercurrent cellulitis.

The last physical condition to which I will call your attention is not so much an indication of the invasion of the psoas muscle as a positive and unfailing sign that we have a pelvic cellulitis to deal with. This is an extension into the cellular tissue of the vaginal wall of the cellulitis of the pelvis, generally by the route of the broad ligament. This can be detected in vaginal exploration as a solid mass between the finger and the lateral pelvic wall, and can be traced upward and lost in the vaginal roof in the lateral fornix. A spur from this vaginal cellulitis sometimes extends partially across the vaginal wall posterior to the vaginal portion of the cervix, but not blending with the uterine wall in the posterior cul-de-sac. This extension into the recto-vaginal septum can be caught up with the index finger in the rectum and the thumb in the vagina, demonstrating absolutely that it is a cellular tissue exudate. Now this is a cellulitis pure and simple; there is no peritoneum or no visceral content here to render the certainty of this demonstration doubtful. It is never a primary condition, but is always secondary to cellulitis of the broad ligament or iliac fossa. It is so easy of detection and verification that the most surprising thing about it is that it has been so long overlooked. I first described and figured it in the Transactions of the American Gynecological Society for 1895. It may also be found described and illustrated in the Transactions of the New York State Medical Association for 1897 and in the BUFFALO MEDICAL JOURNAL for 1895.

The treatment is surgical and represents the best and latest form of intrapelvic surgery. A few years ago, when median abdominal celiotomy was so unreasonably and unscientifically in the ascendant, this condition was attacked in a most dangerous and inefficient manner. The pus accumulation was opened and drained from above, a direction in which it was impossible to shut off the abdominal cavity or efficiently drain the pus accumulation. It may be necessary to open the abdominal cavity in order to be certain of our bearings, but the opening of the abscess cavity and efficient drainage must be made in the direction of its greatest accessibility and the natural gravitation of its contents. Oftentimes these abscesses point above Poupart's ligament, especially when the fossa as well as the psoas is involved and the naturál tendency to open at this point may be taken advantage of, but usually drainage is very tedious and a counter-opening through the vagina is necessary in order to save the patient. The vaginal route is the natural one and any abscess in this region may be opened from this direction. Anteriorly or posteriorly makes but little difference, but I think the anterior cul-de-sac offers some facilities in reaching the fossa that the posterior route does not possess. It is usually necessary to insert drainage. This must never be gauze, for gauze never drains, but it must be a tube, either glass or rubber. A point to bear in mind is the important and practical one—namely, that a patient may go months with such a pus accumulation we have described, possibly with no temperature at all, as in our case, but as soon as the abscess is opened, a serious rise of temperature may occur in from three or four days to a week. A tube allows thorough irrigation of the cavity. I think this was the reason that my case terminated fatally, as irrigation was delayed a little too long. At the same time air must be carefully excluded, as I am satisfied that oxygenation can revitalise sterile pus, or air in contact with the products of the germ renders the ptomaines active. This is my explanation, but it is a clinical fact, no matter how explained, and the only remedy I know of is efficient and thorough irrigation and for which I use sterile water.

404 FAYETTE PARK.

### DISCUSSION.

DR. N. JACOBSON, Syracuse: The case presented is one of great interest to me: first, because of the diagnosis; and, secondly, as to its treatment. Psoas abscess is frequently encountered and we meet it in both sexes and at all ages. The pus collections are seen early in life, when the possibility of its origin, being a cellulitis, cannot be suggested. I must confess that I am not quite convinced that the author of the paper under discussion has properly made his case. I am inclined to view the case rather as one of the many forms in which we encounter pelvic abscess in women and in which neither the psoas nor the iliacus are in any way (at least primarily) involved.

It seems to me that there can be no question as to what we are to include under the designation "psoas abscess." This form of suppurative inflammation, I take it, must in its incipiency and from its very beginning be directly associated with the psoas muscle, either at its origin in the spinal column, or in the investing sheath, or in some other way affecting the muscle or its immediate attachments.

Because a suppurative inflammation has been permitted to exist for a period of time sufficiently long to extend to and secondarily involve the surrounding structures, and amongst them the muscles mentioned, I, for one, cannot conceive that from a pathologic standpoint we can assign to the secondary implication the important place of designating it the primary disease. In my experience it is not an unusual thing, but rather a condition frequently encountered, to meet with pus collections of the nature described, and we have ordinarily found that in their origin they are connected with the tubes or ovaries.

These cases do not usually require abdominal incision, nor do they in the large percentage of cases demand more than vaginal puncture. Taken at a period sufficiently early, this line of procedure is all that is required in most cases. A second operation may be required for the radical removal of the pus sac.

However, while this matter is up, it seems to me that we should lay stress upon the fact that psoas abscess is not the incurable and hopeless condition it was formerly supposed to be. Radically treated, the results should be as good as in most of the other forms of surgical tuberculosis. Some fifteen years ago Treves first undertook his operation for the radical cure of psoas abscess. I have a number of times performed the operation which he describes and feel confident that by either this procedure or by one of the other methods since devised the source of trouble can be reached, whether it be located in one of the bellies of the muscle or at their spinal attachments.

Another thought in the case presented is one that should be emphasised because of its practical importance. Too much stress is laid upon the coëxistence of fever and pus. We frequently meet (and particularly where the pus is locked up in the abdomen) conditions in which there is practically no rise of temperature. I am reminded of one case of psoas abscess in which there was a definite tumor on the left side, in a male, who had not been complaining of localised pain in the region of the tumor, but rather of pain in his right shoulder. At this time he had a temperature of 105°. Under the administration of salicylate of soda, the temperature practically fell to normal, but the man's general condition grew steadily worse. The evidence of pus absorption was manifested in this depressed general condition. At first he refused operation, but finally consenting we opened up the abscess cavity freely. A quart of pus was evacuated, and after two or three months of vigorous treatment he made a complete recovery.

Dr. Ely Van De Warker, Syracuse: Of course, you will recall to mind that I included the psoas abscess due to cellulitis, not as primary abscess of the psoas, but by secondary implication in the line of the least resistance, and having the psoas involved it was a psoas abscess, notwithstanding it was secondary to the primary condition. It deserved that classification, consequently, for that reason furnishing a distinct group of the psoas abscess and one peculiar to women.

---

Bacilli in the Throat.—Hand, of Philadelphia, recommends the solution of nitrate of silver in the strength of 60 grains to the ounce of water to rid the throat of bacilli after the disappearance of diphtheritic membrane. Often after the throat is entirely clear and the patient apparently well, examination shows that bacilli are still present. In such cases quarantine must still be observed. Hand reports very good results from this method, the bacilli usually disappearing within a very few days. The solution should not be used until the membrane has disappeared.—*Cleveland Jour. of Med.*

